# Eco-Bio-Communitarianism and Its Application to Clinical Research, Global Health, Biotechnology, and Environmental Protection

**DOI:** 10.1007/s11673-025-10509-5

**Published:** 2026-02-23

**Authors:** N. S. Munung, G. B. Tangwa

**Affiliations:** 1 Division of Human Genetics, University of Cape Town, Cape Town, 7925 South Africa; 2 Cameroon Bioethics Initiative (CAMBIN), Yaoundé, BP 31489 Cameroon; 3 Department of Philosophy, University of Yaoundé 1, Yaoundé, PB 4559 Cameroon

**Keywords:** Bioethics, Eco-bio-communitarianism, Public health, Cosmic humility, Solidarity

## Abstract

As the world grapples with escalating global health and environmental crises, existing Western bioethics frameworks tend to fall short in addressing the complex interdependencies between human, animal, and ecological health, as well as the diverse cultural and belief systems in many parts of the world. Eco-bio-communitarianism, a bioethics framework informed by an African indigenous worldview, provides a commonsensical, practical, and comprehensive approach that can bring about positive change in global and public health. The framework promotes the principles of planetary kinship, relational interdependence, non-anthropocentricity, eco-bio-centricity, and cosmic humility. Unlike mainstream bioethics, which is frequently driven by individual autonomy and biomedical reductionism, eco-bio-communitarianism situates health within a broader moral ecology that recognizes the interconnectedness of all life forms, including humans, non-human species, ecosystems, and cultures. This paper showcases the practical application and utility of eco-bio-communitarianism through a series of case studies addressing and examining zoonotic disease management, biotechnology governance, and gene therapy. Far from dismissing Western bioethics models, eco-bio-communitarianism invites a pluralistic and decolonial expansion of global bioethics, one that respects cultural specificities and advances ecological responsibility and relational justice, while urging public health practitioners, policymakers, and global health actors to embrace a more inclusive, relational, and culturally attuned perspective to global health and the biomedical sciences.

## Introduction

Wherever humans have lived, they have had to adapt to the specific demands of their ecological niches. This adaptation involves not only biological survival but also changes to social, economic, cultural, and spiritual practices to support human flourishing. Yet, these adaptations, while necessary, can have unintended and adverse consequences for humans, non-human life, and the broader environment (Tangwa 2019). For example, the (re)emergence of infectious diseases such as Zika, Mpox, Ebola, Lassa fever, and COVID-19 has increasingly been linked to ecological disruptions caused by agricultural intensification, urbanization, global travel, and wildlife trade (Karesh et al. 2012; Jones et al. 2008; Cook et al. 2004). Recent global health and policy frameworks, such as One Health, acknowledge the interdependence of human, animal, and environmental health. However, the bioethics paradigms underpinning these frameworks remain either underdeveloped, implicitly anthropocentric, or primarily informed by dominant strands of mainstream bioethics.

Entrenched largely in mid-twentieth century Western liberal thought, mainstream bioethics frameworks tends to privilege individual autonomy and biomedical interventions while limiting engagement with the ecological, cultural, or cosmological dimensions of health. Nevertheless, some Western thinkers have attempted to expand this scope. For example, Aldo Leopold’s “The Land Ethic” (Leopold 2017), Van Rensselaer Potter’s *Bioethics: Bridge to the Future* (Potter 1971), and Holmes Rolston III’s *Environmental Ethics* (Rolston 1988) each emphasize the interdependence of human health and the rest of the natural world. These works critique reductionist approaches to human health and the biosciences. In doing so, they also challenge the anthropocentric assumptions that underpin biomedicine more broadly. These contributions have enriched Western bioethics but are largely compartmentalized within disciplinary silos or fail to account sufficiently for cultural pluralism.

Addressing contemporary global health challenges requires epistemic humility and a pluralistic ethical perspective, one that can be advanced through cross-cultural engagement and inter-philosophies dialogue (Benatar, Daibes, and Tomsons 2021). African philosophies and ethics, such as *Ubuntu* or *Wir dze wir bih wir* (Ramose 1999; Tangwa 2010; Chuwa 2014), and other indigenous knowledge systems (Kimmerer and Artelle 2024), though not always codified into formal bioethics theories, can also provide principled guidance for contemporary global health challenges. To this end, we revisit eco-bio-communitarianism, a normative framework informed by certain African traditions, to explore its relevance for global health ethics. Eco-bio-communitarianism rejects the binary of human versus nature, and instead centres interdependence among humans, non-human species, ecosystems, and metaphysical forces (Munung and Tangwa 2025; Tangwa 1996). Unlike Western communitarianism or environmental ethics, it is grounded in ontologies of relationality, non-anthropocentrism, cosmic humility, and the co-flourishing of all forms of life (Table 1).

**Table 1:** Summary of the principles of eco-bio-communitarianism

**Principle**	**Description**	**Example of applications in global public health and biomedical research**
Planetary kinship	Mutual respect and a sense of responsibility towards all living things (humans, animals, plants), and the environment.	Implementing One Health strategies to prevent and control zoonotic diseases. Developing planetary health frameworks that integrate environmental sustainability with human health policies.
Cosmic humility	Recognizing that humans, with our limited lifespans and perspectives, are just a small part of the universe.	The regulation and practice of gene editing (e.g., CRISPR-Cas9) should acknowledge the limits of human knowledge, how gene manipulation can affect perceptions of identity in humans (as individuals and parts of communities), and certain cultural views about being and belonging.
Non-anthropocentricity	Recognizing the intrinsic value of all living and non-living entities, rather than viewing humans as superior beings with a right to exploit the rest of nature.	Developing biomedical research policies that respect biodiversity and prevent the bioprospecting and genetic exploitation of indigenous plants and animals.
African communitarianism	Emphasizing the primacy of the community over the individual and promoting collective responsibility in decision-making.	Implementing public health strategies based on traditional knowledge systems and practices that do not harm human social structures.
Solidarity and reciprocity	Mutual support and shared human responsibility for all earthly creatures and ecosystem preservation.	Policies that protect indigenous knowledges related to medicinal plants and genetic resources from exploitation by pharmaceutical and biotech companies.
Eco-bio-centricity	Placing equal importance on the health of ecosystems, animals, and humans.	Integrating wildlife conservation efforts into global health programs to address zoonotic disease transmission. Encouraging eco-friendly clinical trials that minimise environmental harm and reduce carbon footprints in global health initiatives.

This paper proceeds in two parts. First, we revisit the theoretical foundations of eco-bio-communitarianism and contrast them with similar bioethics frameworks informed by Western worldviews. Secondly, we apply this lens to pressing global health issues, such as zoonotic disease control, biotechnology governance, and clinical trials, and demonstrate how it aligns with—and enriches—initiatives such as One Health and Environmental Ethics. In doing so, we neither seek to dismiss or disparage Western bioethics theories, rather we expand the global bioethics discourse by highlighting how African contributions that have long emphasized the interconnectedness of human, non-human animals, plants, environmental, and cosmic well-being, can respond to some emerging ethical challenges in the biomedical sciences and global health.

## Revisiting the Theoretical Foundations of Eco-bio-communitarianism

Since the time of Hippocrates of Cos (460–357 BCE), societies have formulated guidelines to inform a moral approach to medicine, science, and human well-being. In the aftermath of World War II, for example, modern Western bioethics emerged primarily to safeguard individual rights and prevent harm in the biomedical sciences. However, the etymology of “bioethics”—from the Ancient Greek words *bios* (life) and *ethos* (moral character)—suggests a broader scope for the discipline than that offered by mainstream interpretations and the predominant focus on the four principles of autonomy, beneficence, non-maleficence, and justice in Western bioethics.

In 1927, Fritz Jahr introduced “Bio-Ethik” as a moral framework inclusive of plants and animals (Sass 2007; Jahr 1927). Other scholarship foundational for Western bioethics, such as “The Land Ethic”, *Environmental Ethics*, and works on social-ecological governance (Leopold 2017; Janssen and Ostrom 2006; Potter 1971), similarly sought to transcend narrow biomedical boundaries and reductionist thinking in biomedicine by stressing the importance of human–environmental relational stewardship. Despite differences in methods and emphasis, these approaches converge on the intrinsic value of ecosystems, the moral significance of non-human life, and the necessity of reciprocal relationships within the biotic community. In this light, they resonate with eco-bio-communitarianism, a bioethics framework informed by an African indigenous worldview and related practices.

Coined by Tangwa (1996), eco-bio-communitarianism responds to the limitations of anthropocentric and discipline-specific bioethics paradigms. It draws heavily from *Wir dze wir bih wir*, the worldview of the Nso’ people of the Northwestern Grassfields of Cameroon. Nso’ culture and traditions resist rigid separations between individual and community, nature and culture, or body and spirit. Eco-bio-communitarianism affirms that human well-being is inseparable from the flourishing of ecosystems and spiritual cosmologies. Unlike mainstream bioethics, which is informed by so-called principlism, eco-bio-communitarianism derives moral obligations from lived relationships between people, species, land, and ancestors, emphasizing the notions of non-anthropocentricity, cosmic humility, planetary kinship, solidarity, and African communitarianism (Table 1).

Eco-bio-communitarianism shares thematic affinities with environmental justice, One Health, and Western communitarian ethics. However, it is distinctive in scope and ontology (Table 2). Importantly, eco-bio-communitarianism is not a wholesale rejection of Western bioethics or environmental thought. Rather, it expands the field by contributing an African perspective with which to reorient our moral gaze in global health. It does not discard principles like autonomy or justice, it situates them within broader obligations to co-flourish with all forms of life, human and non-human, and the environment. The African contribution here is not only cultural or contextual; it is philosophical and actionable. It invites a rethinking of foundational assumptions about the human-nature divide and the narrow framing of health as the absence of disease within the human body. By revisiting the theoretical foundations of eco-bio-communitarianism, this paper provides a lens through which researchers, public health practitioners, and policymakers can adopt a more holistic approach to global health challenges.

**Table 2:** Comparison of eco-bio-communitarianism and related concepts

**Dimension**	**Eco-bio-communitarianism**	**Environmental justice**	**One Health**	**Western environmental ethics**	**Western communitarian bioethics**
Core worldview	Interdependence between humans, non-humans, land, and spirits in African metaphysics.	Human-centred focus on equity in the distribution of environmental harms and benefits.	Integrated view of human, animal, and environmental health.	Nature possesses intrinsic moral value.	Prioritizes community over individual; informed by liberal political theory.
Moral orientation	Holistic relational ethics of reciprocity, solidarity, and cosmic humility.	Rights-based justice and policy redress.	Systems thinking for prevention and risk reduction.	Moral duty to protect nature and future generations.	Commitment to shared norms and public good.
Human–nature relationship	No human/nature divide; kinship with all forms of life.	Humans as justice-seeking agents; nature often instrumentalized.	Humans as stewards of ecosystem health.	Humans as members of the biotic community.	Nature is peripheral; ethics focused on human community.
Epistemological foundations	Indigenous cosmologies, oral traditions, spiritual ecology.	Legal, political, and activist frameworks.	Biomedical science, ecology, and systems theory.	Western ecological philosophy.	Liberal moral theory and communitarian political traditions.
Scope of ethical concern	Humans, ecosystems, and metaphysical realms.	Marginalised human communities and environmental harm.	Primarily human and animal health; some environmental concerns.	Ecosystems, species, and future generations.	Human community, public health, and social cohesion.
Distinctive value	Non-anthropocentric and holistic.	Strong equity focus and policy leverage.	Multisectoral and cross-disciplinary.	Emphasis on the intrinsic worth of non-human life.	Civic virtue.
Key limitations	Requires intercultural translation; risks essentialism.	Largely Anthropocentric.	Cultural insensitivity.	Philosophically too abstract.	Minimal engagement with ecological or non-human concerns.

## Zoonotic Diseases and Public Health Ethics: Integrating Cultural and Moral Ecologies

Zoonotic diseases such as Ebola, Mpox, Lassa fever, and COVID-19 have revealed how entangled human health is with animal populations, environmental change, and cultural practices (Rulli et al. 2017; Wang et al. 2021; Morand and Lajaunie 2021; Saha, Ghatak, and NS 2022). The One Health framework, developed in response to these complex interactions, promotes interdisciplinary collaboration across human health, veterinary medicine, and environmental science (Gruetzmacher et al. 2021). However, it fails to meaningfully engage with local belief systems, cultural taboos, and spiritual worldviews. Yet these factors inform how communities understand and respond to illness and disease. To be effective, applications of One Health must consider how the legacy of Western exploration, proselytization, exploitation, and colonization of Africa, Asia, and Indigenous populations in Western countries, has shaped how people in these regions perceive scientific recommendations and health and environmental policies from the West, especially concerning land use, cultural/behavioural changes, and biomedical experimentation (Tangwa et al. 2019; Kimmerer and Artelle 2024; Sakamoto 2004).

Consider a hypothetical outbreak of Mpox in rural Cameroon (Appendix 1). While a One Health response might prioritize disease containment through surveillance and vaccination, such measures alone are not sufficient. Among many Indigenous communities in Cameroon, certain animals and plants carry spiritual significance or are subject to taboos (Bobo, Aghomo, and Ntumwel 2015; Nkenkuh, Ambebe, and Awazi 2025). Hence, hunting these animals is traditionally restricted or ritualized, and violations are believed to cause spiritual imbalance in society or disease. Similarly, many African communities restrict access to sacred forests for activities that disrespect forests, like lumbering, harvesting, hunting, hiking, or any activity which involves excessive noise (Sinthumule 2024). These taboos reflect an embedded moral ecology, a relational ethic that governs human–animal–spirit interactions. However, over the past decades, these worldviews and practices, once grounded in reverence and restraint, have come under pressure from commercial logging, wildlife trafficking, and an increased demand for bushmeat (Duda 2017; Ngoufo et al. 2014). These shifts, along with the cosmologies that guide them, are largely absent from official public health discourse and policies.

From a One Health perspective, zoonotic disease control typically involves ecological monitoring and risk reduction through wildlife management and vaccination. This approach treats forests and animal populations as passive vectors of disease rather than as spiritually inhabited, morally governed landscapes. By contrast, eco-bio-communitarianism reframes zoonotic outbreaks not only as public health crises but as ruptures within a web of relationships among humans, non-humans, ecosystems, and ancestors. From this standpoint, restoring health therefore requires both biomedical intervention and the repair of relational, cultural, and spiritual bonds. While this approach aligns with One Health, it significantly expands the moral and practical scope of zoonotic disease ethics (Table 3).

**Table 3:** Eco-bio-communitarianism vs One Health for the management of zoonotic diseases

**Framework**	**Focus**	**Ethical limitation**	**Contribution of eco-bio-communitarianism**
One Health	Cross-sectoral integration of human, animal, and ecosystem health.	Technocratic; weak on cultural, spiritual, and moral dimensions.	Calls for relational respect, spiritual diplomacy, and ecological humility.
Environmental justice	Protection of human rights and equitable participation in decision-making.	Anthropocentric; limited inclusion of non-human moral obligations.	Expands justice to ecosystems, animals, and spiritual custodians.
Eco-bio-communitarianism	Interdependence of humans, animals, land, and metaphysical forces.	Difficult to operationalize within current global health norms.	Embeds disease control in a moral ecology of reciprocity, responsibility, and co-existence.

An eco-bio-communitarian approach will favour collaborative efforts in which public health practitioners, policymakers, researchers, and communities work together to develop strategies for mitigating zoonotic spill overs. These strategies would incorporate indigenous knowledge systems on land use, animal interactions, and encroachment into sacred habitats. In this light, key public health strategies would complement biomedical tools with culturally informed actions—such as halting bushmeat trade and limiting deforestation—not just to reduce spill over but to honour the intrinsic dignity of non-human life. This approach will also require spiritual diplomacy, including measures such as consulting traditional custodians before entering sacred forests and trapping animals for scientific testing. Equally, public health surveillance would integrate indigenous beliefs into early-warning systems and risk communication for mPox. This approach does not reject scientific knowledge but insists that disease management should be embedded within the moral ecologies that determine how communities live, relate, and heal.

## Biotechnology Governance Through the Lens of Eco-bio-communitarianism

Human tinkering with nature, which began with agriculture about ten millennia ago, has evolved significantly through modern biotechnology. Today, the human impulse to manipulate nature, once limited to practices like selective breeding and traditional agriculture, have expanded dramatically with the advent of advanced biotechnologies such as CRISPR-Cas9, synthetic biology, and genetically modified organisms (GMOs). While these technologies hold significant promise in addressing food insecurity, disease, and environmental degradation across Africa (Doudna 2020; Ndudzo et al. 2024; Matinvafa et al. 2023), their governance is mostly framed through technocratic and liberal-individualist paradigms. For example, regulatory approaches typically emphasize biosafety, informed consent, and intellectual property rights; all important considerations but largely embedded in anthropocentric and utilitarian frameworks (Tangwa 2010).

Consider a second hypothetical case, involving the introduction of genetically modified (GM) traditional leafy vegetables in the Eastern Cape, South Africa (Appendix 2). A multinational biotechnology corporation, in partnership with government agencies, distributes GM seed varieties of the vegetable to smallholder farmers in a region historically reliant on heirloom seeds and community-based seed-sharing rituals. While bioethicists and regulators framed the intervention in terms of biosafety, market access, and intellectual property compliance, civil society groups and local leaders raised concerns. They argued that commercial agricultural companies label indigenous varieties of the vegetales as “weeds” thereby legitimizing their displacement. This process was understood not as agronomic substitution but as an erosion of seed sovereignty. The is replacement was seen as a threat to agrobiodiversity and a violation of sacred obligations to land, ancestors, and future generations, since the introduction of GM seeds may diminish the cultivation and consumption of the indigenous variety.

In many African and other indigenous cultures, seeds and local foods are not just biological or proprietary materials; they are entwined with cultural identity, ancestral memory, and spiritual stewardship (Maseko et al. 2017; Dweba and Mearns 2011). Farming practices are embedded in traditional systems that view land, plants, and human life as mutually sustaining. However, mainstream biotechnology governance in Africa rarely engages with these dimensions, operating under the assumption that scientific innovation is universally beneficial. Regulatory frameworks focus on biosafety, consumer rights, and food security, but rarely engage with indigenous value systems, communal seed practices, or the spiritual dimensions of farming. This case illustrates the limitations of conventional technocratic or rights-based bioethics frameworks in addressing the full range of ethical questions raised by biotechnology.

From an eco-bio-communitarian perspective, biotechnology must be governed not only by scientific standards and commercial profitability but also by moral obligations to land and community. Eco-bio-communitarianism offers three key contributions to biotechnology governance. Firstly, communities are not passive recipients of technology, but moral agents embedded in a wider web of life. The governance of GMOs needs to account for concerns from communities around agricultural practices and cultures, not just individual choice or expert panels. Secondly, epistemic humility is required. Human knowledge of genetic complexity remains partial. Eco-bio-communitarianism calls for precaution as a scientific principle and a form of humility, and also advocates for the inclusion of traditional knowledge holders alongside scientific experts in biotechnology deliberations. Thirdly, solidarity and intergenerational responsibility are expected from all biotechnology stakeholders. Decisions on the unintended ecological, cultural, and economic impacts of biotechnological change should be made not only with present utility in mind, but also with reverence for future generations and the integrity of communal life. Technology must serve life rather than efficiency alone; it's developers must be accountable to the communities it affects, not only to markets or regulatory thresholds Table 4.

**Table 4:** Comparing ethical frameworks for the governance of biotechnology

**Framework**	**Focus**	**Limitations in biotechnology governance**	**What Eco-bio-communitarianism adds**
Environmental justice	Equity in distribution of risks and benefits; participatory governance.	Tends to be anthropocentric; limited attention to non-human factors.	Situates biotechnology in moral ecologies.
Western environmental ethics	Concern for biodiversity, ecological integrity, and precaution.	Often abstract and eco-centric; weak on cultural and community-specific ethics.	Adds cultural specificity, ancestral obligations, and Afro-communitarian cosmologies.
Eco-bio-communitarianism	Interdependence of all life forms; solidarity, humility, and spiritual responsibility.	Less formalized in law/policy; hard to integrate into existing regulatory systems.	Offers a culturally grounded ethical system to assess the relational, spiritual, and ecological impacts of biotechnology.

## Gene Therapy, Consent, and Eco-bio-communitarianism

Cutting-edge biotechnologies such as gene editing hold great promise for addressing inherited conditions like sickle cell disease (SCD), which disproportionately affects populations in Africa (Johnson 2023). While the implementation of such interventions may satisfy international standards of informed consent and ethics and regulatory compliance, they may fail to engage with the communal and epistemic frameworks through which genetics is understood in African contexts. Gene therapy trials in Africa would likely raise questions that transcend the procedural boundaries of mainstream biomedical ethics (Munung et al. 2024). These include not only what can be done, but how, by whom, and with whom. Ethical scrutiny limited to autonomy, beneficence, and distributive justice may fail to recognize the moral weight of bodily substances and genes in African settings and how scientific research on gene manipulation may influence public trust in science.

Consider a hypothetical case of a multinational research consortium initiating a gene-editing trial for SCD in a teaching hospital in the Southwestern region of Nigeria (Appendix 3). The trial requires blood samples to be collected from participants, processed in European laboratories, and returned for reinfusion following gene editing. While the study complies with all international and national ethics protocols, including informed consent, counselling, and confidentiality agreements, tensions arise from some members of the public, traditional healers, religious leaders, family members of trial participants, and traditional rulers. These individuals do not express concerns about the CRISPR technology itself, but what it represents. In many African settings, blood and genetic material are seen as repositories of identity, ancestry, and spiritual connection. The idea of blood leaving the homeland, being “altered” abroad, and returned may trigger fears of alienation from ancestral lineage. These concerns, while difficult to incorporate within mainstream ethics frameworks, are salient within local moral systems.

From an eco-bio-communitarian perspective, this is not only a question of risk or individual autonomy but one of moral ontology. In many African societies, personhood is relational, constituted through ties to family, land, ancestors, and community. Decisions about genetic modification, be the somatic or germline, may be perceived as implicating not only the individual research participant but the collective community and their belief systems. In this context, cosmic humility (Table 2) is important. Scientific interventions should be approached not only with caution but with reverence for the complex, interconnected dimensions of the lives they affect. This approach proposes several ethical shifts. The first is from individual consent to collective discernment. Gene therapy trials may require broader community engagement, including consultation with elders, traditional custodians, and spiritual leaders. The principle of cosmic humility reminds researchers of the limits of human knowledge and urges caution in introducing new medical interventions, particularly biotechnological and gene-editing therapies whose long-term effects remain uncertain. The second is a shift from procedural ethics to moral ecology. Ethics review committees/boards should also consider if the gene editing align with local understandings and belief systems.

## Discussion

Bioethics discourses in the Western world are undeniably robust, elaborate, and continually evolving in response to advancements in science, technology, and global health. However, the solutions to addressing the intertwined environmental and health crises that we face may not solely lie within Western biomedical and ethics frameworks. Instead, they may be found in the wisdom of African and other Indigenous philosophies that centre relationality and harmony; and that balance and acknowledge not only the needs of humans but also those of non-humans and the broader web of life.

Contemporary global health challenges demand ethical frameworks that go beyond the anthropocentric, fragmented, and disciplinary boundaries of mainstream bioethics. While One Health, environmental justice, and Western communitarianism provide partial responses to the complexities of planetary health, they lack the ontological and cultural depth necessary to address the relational, spiritual, and ecological entanglements that shape health and well-being in many parts of the world. This disconnect limits the relevance and effectiveness of global bioethics in African and other contexts where the integration of human, environmental, and spiritual dimensions of health is paramount. As such, the failure to engage with these non-human-centric concerns risks overlooking critical ethical considerations and mitigating the overall effectiveness of health interventions.

Eco-bio-communitarianism emphasizes relational interdependence, non-anthropocentricity, cosmic humility, and planetary kinship not only toward human beings but also toward non-human life, land, spirits, and future generations. It embeds ethics within indigenous African philosophies that prioritize solidarity over exploitation, cosmic humility over technological dominance, and moral cohabitation over extractive development practices. This expanded ethics horizon provides a more holistic foundation for addressing complex health and environmental challenges. The African Union Agenda 2063, which envisions a healthy and thriving continent with strong cultural values and environmental stewardship, is, in its conceptual dimension, a natural complement to eco-bio-communitarianism.

Eco-bio-communitarianism is therefore more than a philosophical bioethics theory. It is a practical and normative resource for the governance of global and public health. Admittedly from a position of relative weakness and marginalization, it dares to invite global bioethics to deign to listen, learn, and evolve by engaging African philosophical traditions as equal and vital contributors to the international discourse on global health, scientific research, and environmental sustainability. It challenges global health actors, health research promoters, funders, and ethics committees, to acknowledge indigenous knowledges and practices on health and disease and to integrate them into policy formation. In an era marked by wars, epidemics, climate crises, increasing zoonotic spill overs, and rapid biomedical innovations, eco-bio-communitarianism calls for a renewed commitment to cosmic humility, interdependence, and collective responsibility toward all forms of life.

## Limitations of Eco-bio-communitarianism

While eco-bio-communitarianism presents a compelling bioethics framework informed by African indigenous worldviews, it is not without its own limitations. First, its contextual and culturally embedded nature may pose challenges for its application in pluralistic or highly-secular societies. This is true even within Africa, where traditional ways of life and living, as well as collective moral authority for non-human life and the environment, are not widely-shared or legible within policy and legal frameworks. This may make its translation difficult in global health governance structures without significant intercultural dialogue and adaptation. Second, there is a risk of essentializing African thought if eco-bio-communitarianism is presented as a rigid, monolithic worldview. African cultures, despite their remarkable communalities and similarities, are diverse, with varying cultural systems, spiritual cosmologies, and details of relationships to the environment. To avoid over-generalization, any practical application of eco-bio-communitarianism must remain attentive to local variations and specific lived realities. Finally, eco-bio-communitarianism requires institutional and epistemic space to be effectively integrated into mainstream ethics discourse and health governance. This entails the Herculean task of challenging the dominant Euro-American epistemological assumptions that inform and underpin current-day global bioethics and creating genuine opportunities for intercultural deliberations. However, these limitations should be viewed not as disqualifying but as invitations for reflexivity, dialogue, and contextual adaptation.

## Data Availability

Not applicable.

## Appendix 1: Mpox and Cultural Taboos in Rural Cameroon (Hypothetical Case Study)

In Bikum, a village in the East Region of Cameroon, a recent wave of forest clearing for large-scale commercial cocoa farming brought villagers into more frequent contact with wildlife such as squirrels, pangolins, and monkeys. One day, several villagers reported flu-like symptoms and rashes. A young hunter who had encountered, killed, and prepared a monkey for bushmeat developed flu-like symptoms and a characteristic rash. Within two weeks, over a dozen villagers reported similar symptoms, prompting concern about a potential outbreak of Mpox.

Public health officials from the regional capital responded rapidly. Applying the One Health framework, they initiated a vaccination campaign and set up mPox surveillance in Bikum. Community members were advised to avoid bushmeat consumption, and a wildlife monitoring team began trapping and testing local primates to identify and remove “high-risk” species near forest edges. These interventions were met with resistance from some of the villagers.

During a community meeting with the public health team, the villagers accepted the scientific explanation and agreed to comply with the measures. The elders and ritual custodians also informed the public health team that the illness spreading in the village could also have been a consequence of violating long-standing taboos and angering ancestral spirits. In Bikum, monkeys are considered spiritual messengers and hunting them is traditionally forbidden. From their perspective, the outbreak was as much a moral-spiritual breach as it was an epidemiological event.

After the meeting, some community members re-considered their initial position, refused vaccination, and decided not to engage with the public health surveillance efforts. Instead, they turned to traditional healers and organized cleansing rituals to restore balance with the ancestral realm. Though the outbreak was eventually brought under control through combined biomedical and local efforts, the episode left a rift between the community and health authorities and public health officials struggled to reconcile biomedical urgency with the community’s relational and spiritual worldview.

## Appendix 2: Genetically Modified Leafy Vegetables and Seed Sovereignty in the Eastern Cape, South Africa (Hypothetical Case Study)

In a rural region in the Eastern Cape province of South Africa, a multinational agricultural firm launched a programme to introduce a genetically modified (GM) leafy vegetable to smallholder farmers. Extension officers promoted the seed as a solution to food insecurity and emphasized that the modern variety was more nutritious compared to the local indigenous one. The initiative replaced traditional seed varieties with patented GM strains, distributed through public-private partnerships, and branded the local variety as a weed.

While initial uptake was encouraged by promises of higher yields, community elders and local farmers soon expressed concern. For generations, seed-sharing had been embedded in ritual practices and seasonal festivals, marking continuity with ancestors and affirming collective stewardship over the land. The introduction of proprietary seeds disrupted these practices and limited seed-saving.

Community meetings grew contentious when company agronomists dismissed these concerns as “superstitions.” After the first harvest, participating farmers reported higher yields but also unexpected costs for herbicide packages bundled with the seeds. Some community members feared that adopting GM seeds would erode seed sovereignty, disrupt rituals, and increase dependency on outside suppliers. The provincial Department of Agriculture, however, continued with a wider rollout of the GM seeds, while civil society groups petitioned for a moratorium until cultural and ecological impacts were fully discussed.

## Appendix 3: Gene Editing for Sickle Cell Disease in Nigeria (Hypothetical Case Study)

In a university teaching hospital in Southwestern Nigeria, an international clinical trial was launched to test a promising gene-editing therapy for sickle cell disease (SCD). Blood samples from consenting adult participants were collected, shipped to a European lab, edited using CRISPR-Cas9 technology, and then returned for reinfusion. Participants were fully informed, and all legal and ethical standards were met.

During recruitment, families and religious leaders expressed discomfort with the idea of blood “traveling” across continents and its unknown consequences for both participant individuals and future generations. Community engagement teams attempted to bridge the gap but struggled to translate the language of molecular genetics into terms that resonated with the worldviews of the people of Southwestern Nigeria. Despite the scientific rigour of the trial, most families declined to participate.

In response, the hospital advisory board requested a town-hall meeting and proposed that a local priest, pastor, Imam, and a traditional healer (Babalawo) be involved in discussions and project governance. In response, the trial sponsors, citing protocol timelines, declined to convene additional meetings but offered to engage informally by visiting churches, mosques, and public forums to talk about the benefits of the project to SCD patients.

Enrolments slowed. Some parents withdrew their children, preferring established herbal and transfusion therapies. Midway through recruitment, the hospital ethics committee suspended the trial pending dialogue on public perceptions about gene editing and SCD, sample ownership, and post-trial access to treatment.
